# Epidemiological Trends of Dengue Disease in Colombia (2000-2011): A Systematic Review

**DOI:** 10.1371/journal.pntd.0003499

**Published:** 2015-03-19

**Authors:** Luis Angel Villar, Diana Patricia Rojas, Sandra Besada-Lombana, Elsa Sarti

**Affiliations:** 1 Clinical Epidemiology Unit, School of Medicine, Universidad Industrial de Santander, Bucaramanga, Colombia; 2 Clinical Epidemiology Unit, School of Medicine, Universidad Industrial de Santander, Bucaramanga, Colombia; 3 Dengue Medical Direction, Sanofi Pasteur LATAM, Bogotá, Colombia; 4 Epidemiology Direction, Sanofi Pasteur LATAM, México City, Mexico; University of Heidelberg, GERMANY

## Abstract

A systematic literature review was conducted to describe the epidemiology of dengue disease in Colombia. Searches of published literature in epidemiological studies of dengue disease encompassing the terms “dengue”, “epidemiology,” and “Colombia” were conducted. Studies in English or Spanish published between 1 January 2000 and 23 February 2012 were included. The searches identified 225 relevant citations, 30 of which fulfilled the inclusion criteria defined in the review protocol. The epidemiology of dengue disease in Colombia was characterized by a stable “baseline” annual number of dengue fever cases, with major outbreaks in 2001–2003 and 2010. The geographical spread of dengue disease cases showed a steady increase, with most of the country affected by the 2010 outbreak. The majority of dengue disease recorded during the review period was among those <15 years of age. Gaps identified in epidemiological knowledge regarding dengue disease in Colombia may provide several avenues for future research, namely studies of asymptomatic dengue virus infection, primary versus secondary infections, and under-reporting of the disease. Improved understanding of the factors that determine disease expression and enable improvement in disease control and management is also important.

## Author Summary

Dengue disease is caused by one of four serologically related, but antigenically distinct dengue virus serotypes (DENV-1, -2, -3 or -4). It is the most prevalent arthropod-borne viral disease, with a global distribution. Resource-poor countries are particularly vulnerable to transmission of dengue disease and it is present throughout the Americas. Colombia is one of the countries in the Americas most affected by epidemics of dengue disease, which is a significant public health concern. We conducted this systematic literature review to consolidate knowledge regarding the epidemiology of dengue disease in Colombia using well-defined methods to search and identify relevant research, according to predetermined inclusion criteria. The findings reveal that despite vector control measures and constant improvement in diagnosis and clinical management of dengue disease cases by health services, there has been no success in the effective control of the disease. This systematic review identifies important epidemiological characteristics of dengue disease in Colombia, as well as identifying several avenues for future research.

## Introduction

Dengue disease is the most prevalent arthropod-borne viral disease in humans and is caused by any one of four serologically related, but antigenically distinct dengue virus serotypes (DENV-1, -2, -3 or -4). The primary vector for viral transmission is the *Aedes aegypti* (Linnaeus) mosquito. Dengue disease is a rapidly increasing public health priority with a global distribution. Resource-poor countries are particularly vulnerable to transmission of dengue disease [1], and it is present in urban and suburban areas in the Americas, eastern Mediterranean, western Pacific, South-East Asia and mainly rural areas in Africa [2]. Since 1997, symptomatic dengue disease has been categorized by the World Health Organization (WHO) as: undifferentiated fever, dengue fever (DF) and dengue haemorrhagic fever (DHF) [3]. DHF was further classified into four severity grades, with grades III and IV being defined as dengue shock syndrome (DSS). However, a new classification was proposed by the WHO in 2009 based on levels of severity: non-severe dengue disease with or without warning signs, and severe dengue disease, which encompasses DHF and DSS [4].

The WHO estimates that more than 50 million dengue virus infections and 20,000 dengue disease-related deaths occur annually worldwide [2,5]. A recent disease distribution model using a boosted regression tree framework estimated there to be 390 million dengue disease infections in 2010, of which 96 million are clinically apparent [1]. In 2010, the countries of the Americas notified in excess of 1.6 million cases of clinical dengue disease [6]. In Colombia, *Ae*. *aegypti* infestation is widespread and dengue disease is endemic throughout most of the country. Approximately 23 million individuals are considered to be at-risk areas for dengue disease, [7] however, recent reports of dengue disease and *Ae*. *aegypti* at altitudes >1800 metres [8] suggest more people are at-risk.

Colombia has about 46 million inhabitants. Its land area is 1,141,748 km^2^, and three branches of the Andean mountain range dominate its topography [6]. The country can be divided into six geographical regions (Costa Atlantica, Costa Pacifica, Centro Oriente, Centro Occidente, Orinoquia and Amazonia; S1 Fig.), each with distinguishing geographical, climatic and environmental conditions (e.g., altitude, temperature, relative humidity and rainfall characteristics). These regions also have some distinct demographic, socio-economic, political and cultural features. Colombia comprises 32 administrative states called departments that vary considerably in geographical area and size of population. In addition, 10 cities have been designated districts, including Bogotá, Barranquilla, Cartagena and Santa Marta.

Historically, Colombia is one of the countries in the Americas most affected by epidemics of dengue disease [9,10], first recognized as a significant public-health target in the 1950s [11]. In the 1980s, the Colombian National Epidemiological Surveillance System (SIVIGILA) estimated dengue disease incidence was 65.6 per 100,000 population, with no reported severe disease or death [7, 12]. Although the number of annual DF cases ranged from 6,776 to 17,510 during the 1980s [13], there was a clear increase over the decade which continued through the 1990s, with large outbreaks documented in 1990, 1993, and 1998. [7]. The first case of DHF in Colombia was officially notified in December 1989 from the village of Puerto Berrio (Antioquia department) [10,14]. Between 1992 and 1996, more than 1,000 cases of DHF were reported and the frequency of fatal infections increased rapidly [15]. DENV-1 and DENV-2 were the most frequently isolated serotypes in the 1980s and 1990s [12]. DENV-3 is generally believed to have been absent from most of Colombia throughout the 1980s and 1990s [12, 16] re-emerging during the 2002 outbreak [7, 17]. DENV-4 emerged in the early 1980s [12], and cases of DENV-4-related DF have been reported every year since [18].

### Surveillance system

It is mandatory to notify cases of dengue disease to SIVIGILA. Probable and confirmed cases are reported weekly, and cases of serious dengue disease and mortality due to dengue disease are notified immediately. Not all cases of dengue disease are laboratory-confirmed, although all deaths due to dengue disease must be confirmed [19]. The sentinel surveillance system that began in 2000 comprises sentinel institutions that routinely test five patients each week to monitor circulating DENV serotypes. In the case of an outbreak, serological samples are taken from 5% of cases of DF and all cases of serious dengue disease [19, 20]. In 2006, the surveillance system for dengue disease in Colombia began to transition from collective to individual notification. Both systems were used until 2008, after which the collective notification system was no longer used. Discrepancies between local and national data sources may have arisen during the transition period. The newer system generates more data, contributing to an enhanced knowledge of dengue disease in Colombia. Since 2006, the Instituto Nacional de Salud has provided regular disease updates through weekly bulletins and annual reports detailing national and regional incidence information and annual data for dengue-related deaths. Case definitions of dengue disease used in Colombia were changed in January 2010, as the new WHO definitions of dengue disease were adopted [7].

Our systematic literature review describes the epidemiology of dengue disease in Colombia between 1 January 2000 and 23 February 2012 in the context of national and regional (state and district) trends. Incidence (by age and sex), seroprevalence and serotype distribution, and other relevant epidemiological data are described. We also identify gaps in epidemiological knowledge, and aim to provide a basis for defining research priorities for epidemiological studies of the disease and inform evidence-based policies in dengue disease prevention.

## Materials and Methods

A Literature Review Group, comprised of epidemiology and dengue specialists, developed a protocol based on previous literature surveys and analyses [21]. The protocol reflects the preferred reporting items of systematic literature reviews and meta-analyses (PRISMA) guidelines [22] and details well-defined methods to search, identify and select relevant research, and predetermined inclusion criteria to guide study selection. The review protocol was registered on PROSPERO, an international database of prospectively registered systematic reviews in health and social care managed by the Centre for Reviews and Dissemination, University of York on 18 May 2012 (CRD42012002294): http://www.crd.york.ac.uk/PROSPERO/display_record.asp?ID=CRD42012002294/. Papers, theses, dissertations, reports, statistical tables, official web sites and grey materials (e.g., lay publications) were identified using an inclusive search strategy. A heterogeneous group of articles with respect to data selection and classification of cases was anticipated. As these would not be methodologically comparable, a meta-analysis was not planned.

### Search strategy and selection criteria

Searches for epidemiological data relating to dengue disease in Colombia were conducted in a broad range of online sources (S1 Table) between 9 February 2012 and 23 February 2012. Specific search strategies for each electronic database were described with reference to the expanded Medical Subject Headings thesaurus, encompassing the terms ‘dengue’, ‘epidemiology’ and ‘Colombia’. To help increase sensitivity and specificity, combinations of different search strings were used for each electronic database.

Sources were included or excluded according to the criteria defined by the Literature Review Group, which also guided the search and selection process described below, reaching consensus via teleconferences. The criteria allowed for the inclusion of sources containing information related to general epidemiological indicators of dengue disease (incidence and seroprevalence); intensity of dengue epidemics (frequency of hospitalization and severity of attack), populations at increased risk of dengue disease, dengue serotype information, geography of dengue disease and dengue surveillance systems. To reduce selection bias, studies published in English or Spanish between 1 January 2000 and 23 February 2012 were included. This systematic review utilised a protocol common to other reviews in this collection. Within that protocol it was estimated that at least one decade of data would be necessary to provide an accurate image of recent evolution of epidemiology and to observe serotype distribution over time and through several epidemics and to limit any bias that might be introduced by changes in surveillance practices over time; 1 January 2000 was selected as the lower end of the date range for this systematic review due to the sentinel surveillance system in Colombia also began in 2000 and because a summary country surveillance data was presented into the introduction The 23 February 2012 cut-off date reflects when the searches for this systematic review began. For databases that did not allow language and/or date limitations, references not meeting these criteria were deleted manually at the first review stage. No limits by sex, age and ethnicity of study participants or by study type were imposed, although single-case reports and studies that only reported data for the period before 1 January 2000 were excluded. To reduce repetition of published data repeated in meta-analyses or review publications, these duplicate data sets were excluded, unless reporting different outcome measures. Unpublished reports were included if they were identified in one of the sources listed in S1 Table.

Data from other sources were included to complement articles selected in the primary systematic literature review: online reports and guidelines published by relevant organizations; papers and posters from infectious disease, tropical medicine or paediatric conferences; and grey literature were identified through general internet searches (e.g. Google and Yahoo; limited to the first 50 search results). Publications not identified by the approved search strategy and unpublished data sources meeting the inclusion criteria were included if recommended by members of the Literature Review Group.

Following removal of duplicate citations, the Literature Review Group evaluated the list of titles and abstracts, and selected articles considered potentially relevant. A second review was undertaken on the full texts of these documents to select the final list of relevant articles. The Literature Review Group ensured each study complied with the search inclusion and exclusion criteria. Articles and other data sources were not excluded or formally ranked on the basis of the quality of evidence. Although we recognize that assessment of study quality can potentially add value to a systematic literature review, the consensus of the Literature Review Group was that, in this instance, quality assessment would not add value given the expected high proportion of surveillance data among the available data sources and the nature of surveillance data (passive reporting of clinically suspected dengue disease). We therefore retained all available data sources that met our criteria.

The data extraction instrument developed and used for a systematic literature review conducted for Brazil [21] was used to collate and summarize the selected data sources in the form of a series of Excel (Microsoft Corp., Redmond, WA) spreadsheets. Data were extracted into the spreadsheets according to the following categories for descriptive review: incidence, age, sex and serotype distribution, serotype data, seroepidemiology or seasonality and environmental factors, by national or regional groups. Data from literature reviews of previously published peer-reviewed studies and pre-2000 data published within the search period were not extracted. All members of the Literature Review Group had the opportunity to review and analyse the original data sources and extraction tables. No attempt was made to contact researchers for additional information.

## Results

**Fig 1:**
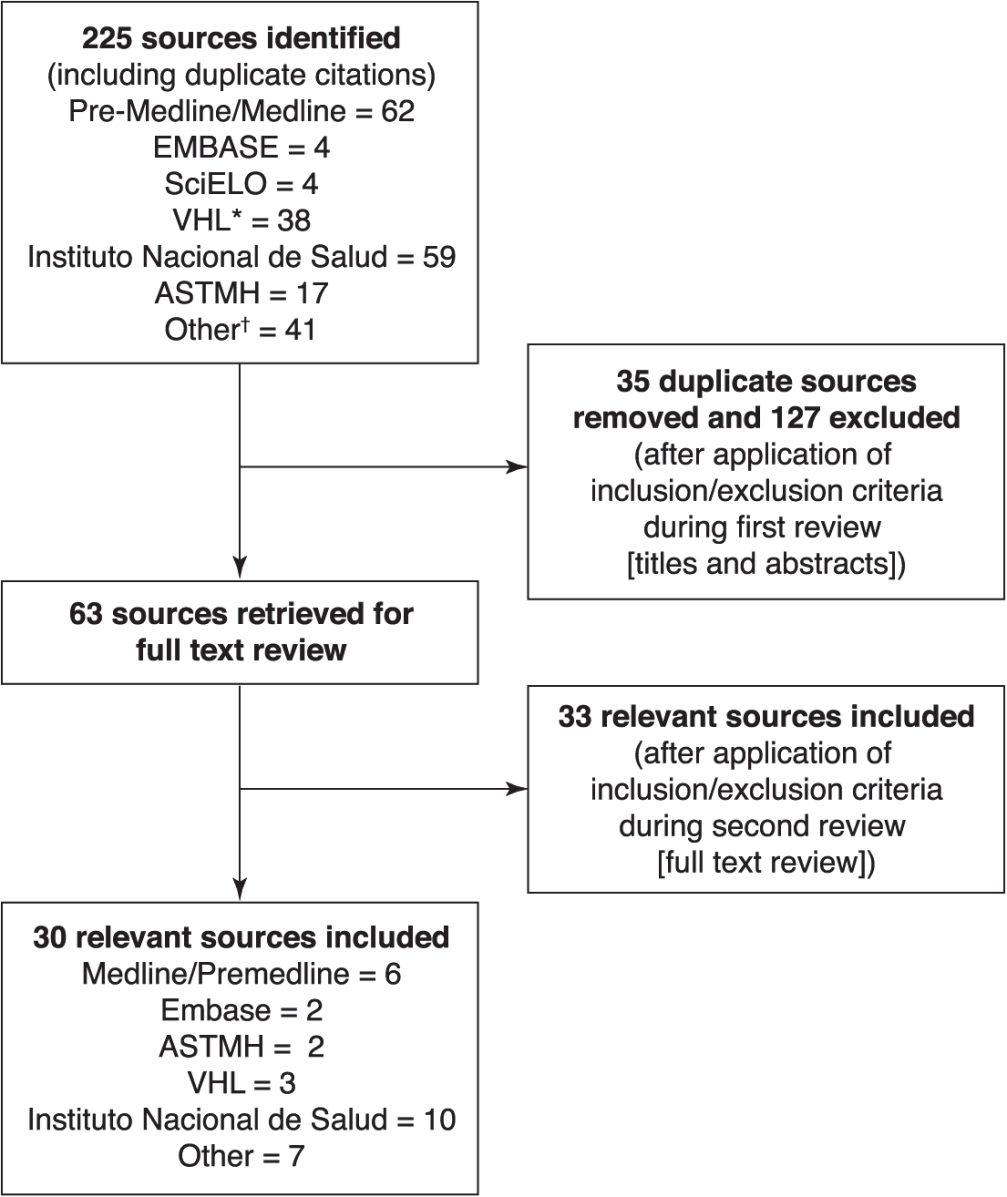
Results of literature search and evaluation of identified studies according to PRISMA. The searches identified 225 relevant citations, 28 of which were dengue-related sources fulfilling the inclusion criteria. All references identified in the on-line database searches were assigned a unique identification number. Following the removal of duplicates and articles that did not satisfy the inclusion criteria from review of the titles and abstracts, the full papers of the first selection of references were retrieved either electronically or in paper form. A further selection was made based on review of the full text of the articles. ASTMH, American Society of Tropical Medicine and Hygiene; EMBASE, Excerpta Medica Database; LILACS, Latin American and Caribbean Health Sciences Database; LRG, Literature Review Group; PAHO, Pan American Health Organization; PRISMA, preferred reporting items of systematic literature reviews and meta-analyses; SciELO, Scientific Electronic Library Online (*includes access to LILACS and PAHO databases); VHL, Virtual Health Library. Other^†^ includes unique references identified from other reference sources detailed in the protocol and LRG bibliographies.

Searches identified 225 relevant citations, following the initial removal of duplicates and papers not matching the study criteria 63 papers were evaluated. Of these 33 were excluded after detailed review of the publication because on further examination data collection occurred outside the search criteria date range, they contained little epidemiological data relevant to the study objectives or because they provided similar but less extensive data to that provided by sources already included and thus provided insufficient information to be included in the review. Some studies were excluded for more than one of these reasons. Consequently, 30 dengue-related sources were included (Fig. 1, S2 Table), of which, 14 and 16 sources were published in English and Spanish, respectively. There were 18 journal articles and three conference presentations/abstracts. The majority of these publications (n = 8) provided analysis of national surveillance data, providing dengue case counts, with some characterization by disease severity, geographic region, and serotype. Six were cross-sectional studies usually limited to specific geographic regions. Only two prospective studies were identified, four studies were phylogenetic studies and one was a disease awareness survey. The remaining 9 sources were recommended and accessed by members of the LRG and comprised surveillance reports, statistical tables (n = 8) and data reported in the text book ‘*Dengue en Colombia*: *epidemiología de la reemergencia a la hiperendemia (Dengue in Colombia*: *epidemiology of hyperendemic re-emergence’* [7].

### National epidemiology

**Fig 2:**
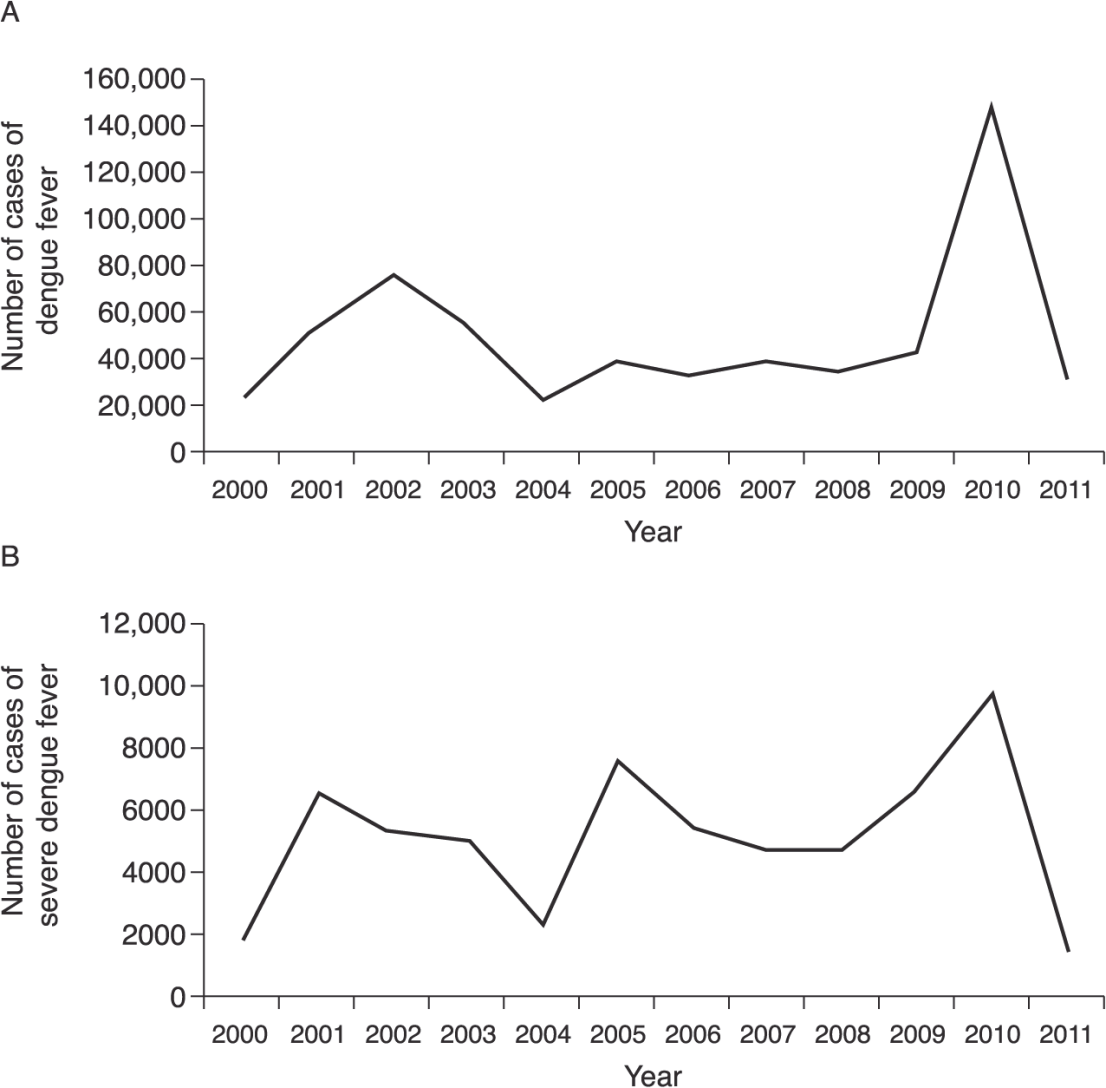
Cases of (A) dengue fever and (B) severe dengue fever in Colombia, 2000–2011 [7]. The epidemiology of dengue disease in Colombia was characterized by fluctuations in the number of DF cases (there was a slight baseline increase over time) with major outbreaks in 2001–2003 and 2010. Widespread dengue disease epidemics were observed during 2001–2003 and 2010. A significant outbreak of dengue disease occurred between 2001 and 2003. The annual number of severe dengue disease was highest in 2010, and lowest in 2011.

Between 2000 and 2011, the annual number of non-severe dengue disease cases reported in nationwide surveillance data ranged between 22,775 (2000) and 147,670 (2010) (Fig. 2) [12, 23].

Widespread dengue disease epidemics were observed during 2001–2003 and 2010. A significant outbreak of dengue disease occurred between 2001 and 2003 **(** Fig. 2), peaking in 2002, when approximately 77,000 non-severe cases of dengue disease were reported (372 cases per 100,000 population) [7, 24]. In this outbreak, the annual number of cases of severe dengue disease peaked in 2001 (approximately 6,600 cases) and 2002 (5,200–5,300 cases) [7, 23]. During the period 2004–2008, the annual number of cases was within the range 22,201–39,814 (Fig. 2) [7, 25, 26]. A slight increase in the number of notified cases of non-severe dengue disease was observed in 2009 [7] (44,412 [26]; 41,819 [27]).

A record number of cases of non-severe dengue disease was reported for 2010 (range: 147,423 [7, 27]–147,670 [22]). The estimated incidence was 577 per 100,000 population) [7, 28] (Fig. 2). Fewer than half of the cases were confirmed using serological or virological tests. Following the 2010 epidemic, the reported number of DF and severe dengue disease cases declined dramatically, resulting in a total of 31,372 DF cases in 2011.

### Severe disease

Across the period 2000–2010, the annual number of severe dengue disease cases reached a maximum of 9,777 (38.3 per 100,000 population) in 2010, and a minimum of 1,383 in 2011 (Fig. 2) [7, 23, 29, 30]. The percentage of dengue disease cases classified as severe (DHF/DSS) changed over time. The percentage of severe cases was lowest in 2011 (4.2%) and highest in 2005 (16.4%) [7, 29]. There was an apparent increase in the proportion of severe cases between 2000 and 2009, whereas the data for 2010 and 2011 suggest a recent decrease[7]. The hospitalization rate for DF cases was 32%, whereas that for severe dengue disease cases was 79% [30], which were not dissimilar to the rates reported for 2009 (31% and 76%, respectively) [26].

### Dengue-related deaths

Compared with data for the 1990s, there was an increase in the number of dengue-related deaths during 2000–2011. A total of 1,040 dengue-related deaths were reported during 2000–2011[7], compared with 439 during 1990–1999 [7]. A total of 217 dengue-related deaths were reported during 2010 [7], which was a considerable increase over the numbers reported in previous years (20–48 annual deaths for 2006–2009). The case fatality rate among patients with severe dengue disease was generally lower during the review period (0.1–5.3% during 2000–2010 compared with 0.4–40% during 1990–1999) [7]. However, the dengue-related case fatality rate (dengue virus infection confirmed by laboratory analysis) in 2010 (2.2%) was the highest since 2002 [7], and increased to 3.1% in 2011 and 3.9% in 2012 (until 23 July) [31, 32].

### Regional epidemiology

Data from the official national reports (Instituto Nacional de Salud) characterize regional variability in the patterns of dengue disease transmission, which differed between regions and even between the departments making up the regions (Table 1).

**Table 1:** Number of reported cases of dengue disease and severe dengue disease by region, 2000–2010.

Year	Costa Atlántica		Centro Oriente		Centro Occidente		Orinoquía		Amazonía		Costa Pacífica	
	Dengue	Severe Dengue	Dengue	Severe Dengue	Dengue	Severe Dengue	Dengue	Severe Dengue	Dengue	Severe Dengue	Dengue	Severe Dengue
2000	5116	342	7453	788	3133	111	1963	143	1409	192	2780	59
2001	7458	3008	**26,110**	**7889**	5871	604	2951	483	1254	350	**8599**	625
2002	**15,332**	486	24,997	1988	**15,563**	**490**	**4927**	**67**	1375	103	**12,140**	1768
2003	4979	145	20,732	3465	**14,347**	**213**	**5493**	**80**	835	35	5010	917
2004	1848	87	9099	1663	4880	92	3110	64	653	82	1826	238
2005	5522	378	11,014	2945	8671	120	**7418**	**181**	587	110	4045	503
2006	7110	607	11,293	3752	5996	164	5372	140	773	179	2095	235
2007	8010	593	12,038	2535	7165	82	6494	533	933	234	3219	567
2008	9632	483	11,471	3096	3808	209	6465	440	839	113	2108	348
2009	5995	293	15,616	4272	2969	122	7250	515	772	82	**10,014**	1189
2010	**12,627**	622	**49,059**	**5414**	**48,561**	**850**	**11,349**	**685**	**2519**	**173**	**20,822**	1718
Total	83,629	7044	198,882	37,807	120,964	3057	62,792	3331	11,949	1653	72,658	8167
Total dengue cases	90,673		236,689		124,021		66,123		13,602		80,825	
Mean incidence (per 100,000 population)	128	119	517	357	197	122	598	607	362	273	173	109
Predominant DENV serotypes	3 (followed by 1, 2 and 4)		1 and 3		3 (followed by 1, 4 and 2)		1 (followed by 3 and 2)		1 and 2		3 (2001−2002); 2 (2003−2010)	
Age groups most affected	<15 years		<15 years		1–14 years		5−14 years (2008: <1 year)		5–14 years and <1 year		5−14 years (2010: <4 years)	

(Outbreak years, characterized by higher than usual numbers of cases in bold) [7].

Most cases of dengue disease occurred in the urban areas of Colombia. Approximately half of all dengue disease cases during the review period were from 18 endemic municipalities. There was an apparent increase during the review period in the geographical area from which dengue disease cases were reported in nationwide surveillance data, with a 90% increase in the number of municipalities between 2000 and 2010 (from 424 to 743) [7]. The incidence of dengue disease was generally low in south and south-east Colombia, attributable to this region of the country having the lowest population density.

The most affected region was Centro Oriente (40% of cases reported during the review period), mainly concentrated in the departments of Santander, Norte de Santander and Huila. Centro Occidente was the second most affected region (20%); for the majority of the review period, most cases in Centro Occidente were in the departments of Quindio and Risaralda, although in 2010, the proportion was largest in the department of Antioquia. Costa Atlantica reported 15% of the cases during the review period, most of which occurred in the departments of Atlantico and Cesar and Barranquilla district. Costa Pacífica region reported 13% of the total cases; Valle del Cauca was the principal department in which dengue disease cases occurred (mainly in 2009 and 2010) [7]. Two regional departments, Vaupés and Amazonas (both in the Amazonía Region), had no dengue disease transmission (despite the presence of the primary vector) until 2009, however, there were reports of dengue disease in both departments during the 2010 outbreak [7].

### Demographic patterns of dengue disease in Colombia

#### Age distribution

**Fig 3:**
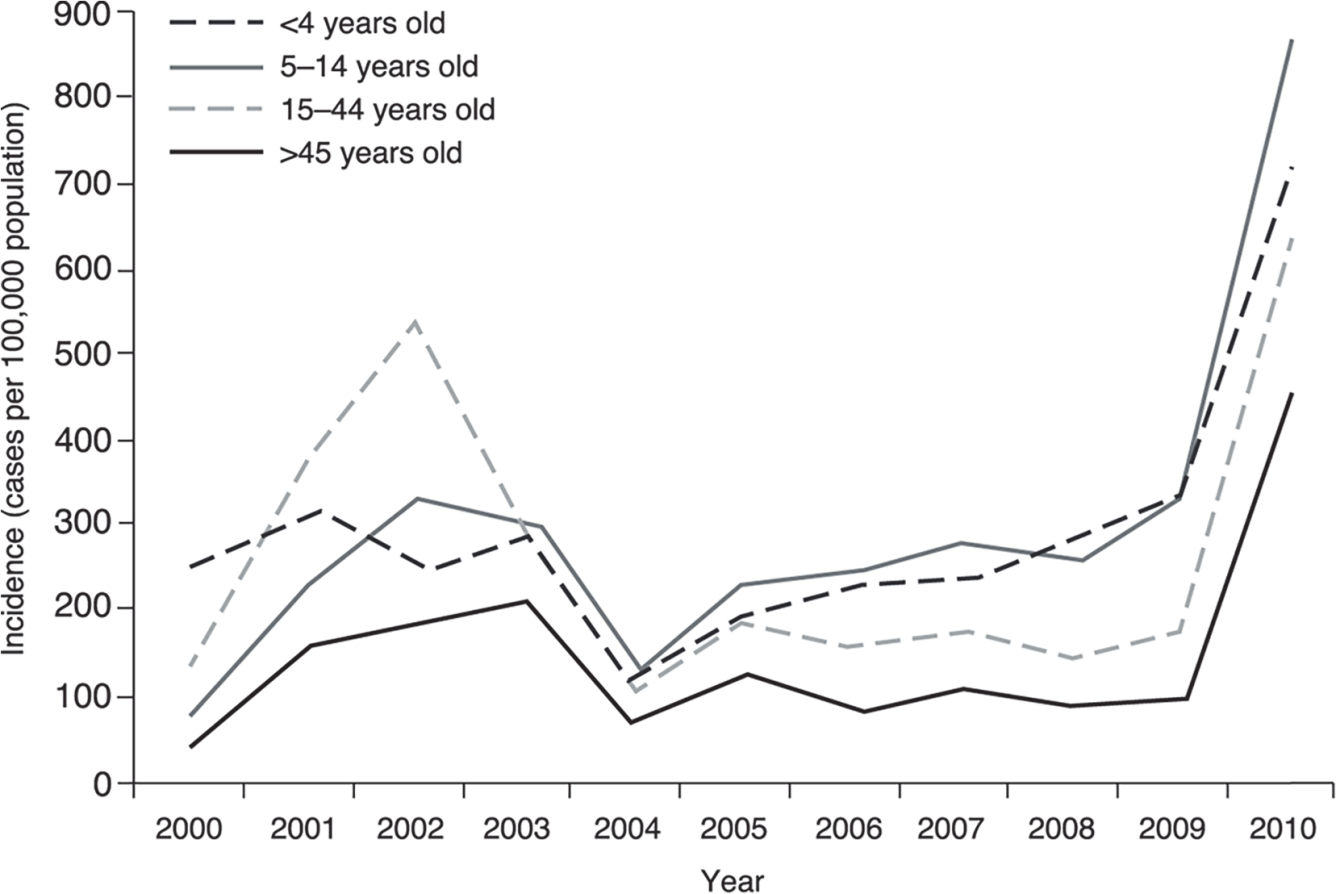
Incidence of reported cases of dengue (per 100,000 population) by age group, 2000–2010 [7]. In the early part of the review period, the highest incidence of dengue disease was in in individuals aged 15–44 years [12]. However, the age distribution of the disease changed and from 2004 to 2010 the highest reported incidence was in the <4-year-old and 5–14-year-old age groups.

From 2000 to 2003, the age distribution of dengue disease was similar to that noted throughout the 1990s, with the highest incidence in individuals 15–44 years of age [12]. However, from 2004 to 2010, the age distribution of the disease changed, and the highest reported incidence was in the <4 years and 5–14 years age groups (Fig. 3) [12, 33].

Regional demographic patterns of dengue disease did not follow precisely the patterns observed at a national level. In a descriptive study, Rodríguez et al. reported data for Palmira (Valle del Cauca) for 2003, showing that the highest incidences of DF (1,163 per 100,000 population) and DHF (27 per 100,000 population) were in individuals 5–14 years of age [33]. The following year (2004), the highest incidence of DF was reported in individuals aged ≥60 years (528 per 100,000 population), followed by children <5 years of age (<1 year: 399 per 100,000 population 100,000; 1–4 years: 341 per 100,000 population), with no cases of DHF reported for these age groups. In contrast, in a virological and serological surveillance study supported by histopathological examination in Guaviare, analysis of sera from 1,049 patients presenting with fever during the first 37 epidemiological weeks of 2004 showed that the highest frequency of dengue disease cases was in the 20–29 year age group [34].

Since 2003, there has been a significant increase in deaths in individuals aged <14 years and >45 years, suggesting a change in the transmission pattern from endemic–epidemic to a hyperendemic pattern. The case fatality rate over that period has generally been highest in the group aged >45 years, except in 2006 when the case fatality rate was highest in the group aged 5–14 years [12].

#### Sex distribution

Reports from nationwide surveillance data of the distribution of DF and severe dengue disease by sex in Colombia over the period 2007–2010 have shown that males and females account for a similar proportion of cases [25, 26, 30]. At a local level, however, some sex differences have been reported. For example, in a descriptive cross-sectional study of individuals with symptoms of fever attending a hospital in Puerto Inírida, Guainía, between November 2007 and February 2008, 1,238 were identified as having probable DF or DHF [35]. Of these patients, 60.8% were female. In contrast, during sentinel surveillance in Guaviare in 2004, 61.6% of confirmed dengue disease cases were in males [34]. In a descriptive, retrospective study, boys and girls accounted for a similar proportions of children <13 years of age (n = 105) admitted to hospital in Neiva (Huila) with DF (12.4%) or DHF (87.6%) in 2004 [36].

#### Dengue virus serotype distribution

For the years when data were available, all DENV serotypes were present in Colombia at some time during the review period (Table 2) [7]. All four DENV serotypes were co-circulating from 2006 to 2010. DENV-3 re-emerged in 2002 after being absent for more than two decades; this serotype was then present every year except 2004. It has been suggested that DENV-3 entered Colombia from Venezuela, with Cucuta as the entrance point [17]. A study evaluating the dynamics of transmission of dengue virus during and after epidemics was conducted between November 2002 and March 2004, and reported the endemic circulation of DENV-3 in south-western Colombia after its re-emergence in the north-western region of the country [37]. Four years after its reappearance in Colombia, DENV-3 was detected in the northern, southern and south-eastern regions of Colombia [36]. The reappearance of DENV-3 coincided with a national epidemic, although the lack of a major increase in the proportion of severe cases suggested no particular association of DENV-3 with severe disease. During the epidemic of 2009–2010, viral isolation and reverse transcriptase-polymerase chain reaction studies were performed on 662 samples at the Instituto Nacional de Salud laboratories [7]. DENV-1 (43.8%) and DENV-2 (40.4%) were the predominant serotypes, followed by DENV-3 (12.5%) and DENV-4 (3.1%).

**Table 2:** Serotype distribution in Colombia: national and regional data from 2000 to 2010.

Year	Region		DENV-1	DENV-2	DENV-3	DENV-4	Source of data: First author, year [Ref]
**2000** *	Regional	Santander	10%	70%	0%	20%	Ocazionez, 2007 [38]
**2000/01**		Santander		48.5%			Ocazionez, 2007 [38]
**2001**	National		Yes	Yes	No	Yes	Padilla, 2012 [7]
	Regional	Santander	4%	40%	36%	20%	Ocazionez, 2007 [38]
		Santander	4%	40%	36%	20%	Ocazionez, 2007 [38]
**2002**	National		Yes	No	Yes	Yes	Padilla, 2012 [7]
	Regional	Santander	6.7%	6.7%	86.6%	0%	Ocazionez, 2007 [38]
		Caquetá	2^†^	0^†^	0^†^	0^†^	Mera, 2003 [24]
		Guaviare	8^†^	5^†^	0^†^	1^†^	Mera, 2003 [24]
		Norte de Santander	0^†^	0^†^	0^†^	1^†^	Mera, 2003 [24]
		Putumayo	5^†^	1^†^	0^†^	0^†^	Mera, 2003 [24]
**2002/03**	Regional	Santander	1.9%	3.8%	94.5%		Ocazionez, 2006 [36]
		1.1.1.1.1.1.1 Valle del Cauca: cities of Cali, Palmira, Tuluá and Buenaventura	18.1%	45.2%	7.8%	28.9%	Méndez, 2006 [37]
**2003**	National		Yes	Yes	Yes	No	Padilla, 2012 [7]
	Regional	Santander	0%	2.7%	97.3%	0%	Ocazionez, 2007 [38]
**2003/04**	Regional	Santander			88.1%		Ocazionez, 2006 [48]
**2004**	National		Yes	Yes	No	Yes	Padilla, 2012 [7]
	Regional	Santander	4.7%	9.5%	75%	14.2%	Ocazionez, 2006 [48]
		Santander	4.8%	9.5%	71.4%	14.3%	Ocazionez, 2007 [38]
**2005**	National		Yes	Yes	Yes	No	Padilla, 2012 [7]
	Regional	Ocaña, Santander	5%	77.5%	12.5%		Rangel, 2008 [49]
**2006**	National		Yes	Yes	Yes	Yes	Padilla, 2012 [7]
	Regional	Ocaña, Santander	57.5%	22.5%	27.5%		Rangel, 2008 [49]
**2007**	National		Yes	Yes	Yes		Padilla, 2012 [7]
	Regional	Santander	36.3%	21.2%	42.2%		Rangel, 2008 [49]
**2007/08**	Regional	Puerto Inirida, Guainia	50%	50%			Rojas-Álvarez, 2008 [35]
**2008**	National		Yes	Yes	Yes	Yes	Padilla, 2012 [7]
	Regional	Santander	85%	10%	5%		Rangel, 2008 [49]
**2009** *	National		Yes	Yes	Yes	Yes	Padilla, 2012 [7]
**2010** *	National		Yes	Yes	Yes	Yes	Padilla, 2012 [7]

National data source: Virology Laboratory, National Institute of Health.

DENV, dengue virus.

*National data unavailable for 2000; regional data unavailable for 2009 and 2010.

^†^Number of positive cases.

Some of the most comprehensive virological and serological regional data for the review period were available for the department of Santander (Table 2) [36, 38, 39], with some data available for other regions. The data from Santander show that changes in relative DENV serotype abundance during this period have been associated with changes in infection pattern and DHF frequency [36, 38]. Dengue disease incidence and serotype data were also reported in the department of Antioquia [40, 41]. The reappearance of DENV-3 appears to have had no impact on the proportion of dengue disease cases classed as severe in Colombia, indicating that this serotype has no significant effect on disease severity [40].

Phylogenetic studies have demonstrated genetic diversity within viruses of the same genotype of DENV-1, DENV-2 and DENV-3. No relationship has been found between DENV variant and clinical disease severity [10, 17, 40]. Regarding genotypes, for DENV-1, the most frequent is the genotype V linage 1 and 2; for DENV-2, currently the American–Asiatic genotype is circulating; for DENV-3, the genotypes III and recently I are the most common; and for DENV-4, the genotype circulating in Colombia corresponds to strains from Indonesia, Tahiti, the Caribbean, and Central and South America [7].

## Discussion

This systematic literature review is one of a series conducted within a similar timeframe in selected countries of the Americas and Asia. The series serves to provide an overview of the changeable epidemiology of DF and severe dengue disease across a broad geographical area over a decade or more.

This review highlights the record number of cases of non-severe dengue disease reported for 2010 in Colombia. There are many complex factors that may contribute to a high incidence of dengue infection. The large majority of dengue disease notifications came from urban areas. High population density and poor infrastructure (e.g., water supplies, sewage systems) encourage standing water, thus providing *Ae*. *aegypti* breeding sites and facilitating the spread of dengue disease in urban areas [42]. Hyperendemicity (the co-circulation of multiple dengue virus serotypes) [43], high temperatures as a result of the El Niño-Southern Oscillation, combined with heavy rains [44], as well as the inadequacies of current methods to reduce dengue transmission [45] also contribute to an increase in incidence of disease owing to an increased frequency of epidemic transmission. Socio-economic factors, including income, may also contribute to high dengue disease endemicity [46]. The recent decrease (2010 and 2011) in the proportion of severe cases could be explained in part by the change in case definitions of dengue disease used in Colombia from January 2010, when the new WHO definitions were adopted in line with global recommendations [7]. Local variations in the distribution of dengue disease between males and females could be due to cultural and social differences or underreported cases [47]. The apparent reduction in the case fatality rate between 2000–2010 may reflect improvements in diagnosis and treatment, however, the reasons for the more recent (2010–2011) increase in case fatality rate are unclear.

Changes in the relative abundance of DENV serotype or genotype may potentially affect dengue virus infection patterns and the frequency of severe disease. For example, although the appearance of DENV-3 was closely associated with the epidemic that lasted from 2001 to 2003, Ospina et al. found that DENV-3 appeared to have no particular association with severe dengue disease [40]. Indeed, there is little robust evidence available to show the impact of DENV serotypes or subtypes on the incidence or severity of dengue disease in Colombia [40].

Some information was either sparse or absent from the selected papers. In particular, there is a lack of information regarding rates of asymptomatic dengue virus infection, primary and secondary dengue virus infections, risk factors for severity and under-reporting of dengue disease in Colombia. These gaps may be addressed by improved surveillance methods for dengue disease. Other data gaps include national and regional data on seroprevalence (laboratory-confirmed cases), the dynamics of dengue disease transmission, and percentages of individuals with dengue virus infection requiring admission to hospital. No studies assessing the sensitivity of the epidemiological surveillance system were identified. In particular, accurate seroprevalence data will improve knowledge regarding the level of transmission and allow more accurate estimations of the transmission rate. No comprehensive data on genotype distribution or change over time were retrieved by this review, and there is a lack of definitive evidence to characterize the links between DENV serotype, genotype or subtype with the incidence or severity of dengue disease in Colombia. Thus, the reasons behind recent epidemics in Colombia are poorly understood. Furthermore, as ethnicity is considered to be a factor that may affect the expression of dengue disease and the risk for severe dengue, more studies on its impact would be beneficial.

The review protocol aimed to minimize potential exclusions of valuable data sources, a factor that is a strength of this systematic review. We not only searched for published articles, but also for relevant books, congress abstracts, theses and dissertations, and unpublished data. However, this systematic literature review is subject to publication bias and the data presented in this report should be interpreted accordingly. A further limitation of this review is that none of the published articles was a population study providing national prevalence data, and consequently it relies heavily on data reported to SIVIGILA. In the years since 2000, national reports of the annual number of cases of dengue disease have been variable in quality, although regular, detailed annual reports over the past 3 years (2009–2011) from the Instituto Nacional de Salud suggest greater vigilance in reporting the disease. The annual reports that have been retrieved show large variations in the number of notified cases for different years.

Shortcomings of the surveillance system have been highlighted, mainly in relation to it being a passive system reliant on notification of clinically apparent disease in humans [37]. As a result, these data may be an under-estimation of the number of cases of dengue disease. The use of other information systems in some studies (e.g., SIS [susceptible-infected-susceptible] model, alert action system) introduces the possibility that not all retrieved data are directly comparable. Clinical symptoms of dengue disease are non-specific and access to dengue diagnostic tests is limited in some parts of the country and, therefore, many cases may go undetected. A correlation has been observed between younger age at presentation of symptoms and a greater need for hospitalization [23].

Despite the availability of the WHO criteria, consistent interpretation of the clinical criteria for dengue disease cannot be guaranteed within surveillance programmes, and classification of cases as severe or non-severe is also liable to vary between physicians. Due to the current passive system of surveillance of dengue disease cases and challenges in diagnosis, official reports of epidemiological data are likely to underestimate the disease burden, and over-estimation is also possible. To improve evaluation of trends in dengue-related morbidity and case fatality, it would be necessary to assess and optimize the sensitivity and specificity of the surveillance system, as well as the classification of severe and lethal cases.

As noted, extensive serological surveys of the seroprevalence of antibodies to dengue virus would be needed to provide a more accurate estimate of the disease burden. However, despite the drawbacks, surveillance data are extremely important for the identification of disease trends, and the available data do indicate trends in the changing nature of dengue disease in Colombia over the review period.

## Conclusions

Dengue disease is a serious public health priority in Colombia and control of the disease has been difficult. The period from 2000 to 2011 has been characterized by a stable ‘baseline’ annual number of DF cases, punctuated by major outbreaks in 2001–2003 and 2010. Between 2000 and 2010, there was a general increase in the annual number of DHF cases, but this was followed by a considerable decrease in 2011. Conversely, the case fatality rate, which was generally lower during 2000–2009 than in the 1990s, began to increase in 2010 and showed a large increase in 2011. The geographical spread of dengue disease cases showed a steady increase between 2000 and 2010, with most of the country affected by the 2010 outbreak. During the review period, the majority of dengue disease cases in Colombia occurred among those <15 years of age, with the highest incidence in 2009 among infants <1 year of age. Overall, there is a lack of definitive evidence to characterize the links between DENV serotype, genotype or subtype with the incidence or severity of dengue disease in Colombia.

## Supporting Information

S1 Checklist PRISMA 2009 checklist. (PDF)

S1 Table Databases searched for citations relating to dengue disease epidemiology in Colombia. (PDF)

S2 Table Table of evidence for sources fulfilling the inclusion and exclusion criteria and included in the data extraction process for the review. (PDF)

S1 Fig Map of Colombia. (PDF)
