# Supplementary material for: Epidemiological Trends of Dengue Disease in Colombia (2000-2011): A Systematic Review
Source: PLoS Negl Trop Dis. 2015 Mar 19;9(3):e0003499. doi: 10.1371/journal.pntd.0003499 (PMC4366106; doi:10.1371/journal.pntd.0003499)
Supplement: S1 Table — (PDF) [file pntd.0003499.s002.pdf]

Table S1. Databases searched for citations relating to dengue disease epidemiology in Colombia.

Database	Website
United States National Library of Medicine and the National Institutes of Health Medical Database (Medline/PubMed)	http://www.ncbi.nlm.nih.gov/pubmed/
Scientific Electronic Library Online (SciELO)	http://www.scielo.org/php/index.php?lang=en
Latin American and Caribbean Health Sciences Database (LILACS) – part of the Virtual Health Library (VHL)	http://lilacs.bvsalud.org/
WHO Library database (WHOLIS)	http://www.who.int/publications/en/
Pan American Health Organization (PAHO) Headquarters Library database	http://new.paho.org/
Instituto Nacional de Salud:<br>SIVIGILA<br>IQEN (Informe Quincenal Epidemiológico Nacional)<br>BES (Boletin Epidemiologico Semanal)	http://www.ins.gov.co/
University websites:<br>Universidad Industrial de Santander<br>Universidad de los Andes<br>Universidad Javeriana<br><br>Universidad del Rosario<br>Universidad de Antioquia<br>Universidad del Valle<br>Universidad Nacional de Colombia<br>Biblioteca Luis Ángel Arango<br>Hemeroteca Nacional: Carlos Lleras y Manuel del Socorro Rodríguez<br>Colciencias<br>Geographic Institute Agustin Codazzi	 http://www.uis.edu.co/webUIS/es/index.jsp.<br>http://www.uniandes.edu.co/<br>http://puj-portal.javeriana.edu.co/portal/page/portal/ PORTAL_VERSION_2009_2010/es_inicio<br>http://www.urosario.edu.co/<br>http://www.udea.edu.co/portal/page/portal/portal<br>http://www.univalle.edu.co/english/<br>http://www.unal.edu.co/<br>http://www.banrepcultural.org/blaa<br>http://vlex.com.co/tags/hemeroteca-nacional-colombia-3619118<br>http://www.colciencias.gov.co/<br>http://www.igac.gov.co/igac
