# Supplementary material for: Epidemiological Trends of Dengue Disease in Colombia (2000-2011): A Systematic Review
Source: PLoS Negl Trop Dis. 2015 Mar 19;9(3):e0003499. doi: 10.1371/journal.pntd.0003499 (PMC4366106; doi:10.1371/journal.pntd.0003499)
Supplement: S2 Table — (PDF) [file pntd.0003499.s003.pdf]

Table S2. Table of evidence for sources fulfilling the inclusion and exclusion criteria and included in the data extraction process for the review

Citation	Region/geographical area	Study design/data gathering method	Data period: Date range/year	If study: No. patients/ population studied (M:F)	Age range	Summary of data presentation or results/conclusion
Padilla et al., 2012 [7]	Nationwide data	Surveillance data analysis	30 years	n/a	n/a	Comprehensive analysis of Dengue in Colombia over 30 years. From 2000 onwards, dengue data reported to SIVIGILA (National surveillance system) was used.
Rodriguez et al., 2005 [8]	Cáqueza, Cundinamarca department	Cross-sectional study	2004 (4 weeks: June–July)	253 (24.4%:75.6%)	6–85y (mean 39.6y)	Household infestation index was 32,9 %. The study showed that dengue's urban vector can reach dangerous levels of infestation above 1 700 m above sea level; 28.8 % of those positive for Ig-G had symptoms of dengue during the last year. People infected on the same town block was significantly related to the probability of being infected by dengue (OR: 1.06; 95% CI=1.04; 1.07; $p < 0.001$).
Mendez et al., 2012 [10]	n/a	Phylogenetic study	No dates given	n/a	n/a	Clinical samples were collected between 1982 and 2010 from different localities. The oldest isolates belonged to the American genotype (subtype V), but the strains collected since 1990 represent the American/Asian genotype (subtype IIIb).
Suarez et al., 2005 [11]	Villavicencio, south-east Colombia	Three data gathering methods used: semi-structured interviews, focus group discussions and direct observation	2001–2002	n/a	n/a	It was observed from individuals' therapeutic itineraries and their perception of disease that public policies advertised in booklets and flyers and on television differ radically from people's everyday reality. This difference influences the success or failure of these policies.

Citation	Region/geographical area	Study design/data gathering method	Data period: Date range/year	If study: No. patients/ population studied (M:F)	Age range	Summary of data presentation or results/conclusion
Padilla et al., 2010 [12]	Nationwide data	Surveillance data analysis<br><br>[Conference presentation abstract]	1980–2009	n/a	n/a	Dengue cases reported to SIVIGILA (National surveillance system) were analyzed from three periods: 1980- 1989, 1990-1999 and 2000-2009. Over the past three decades an increase in reported cases was observed. The data reported suggest that Colombia is trending toward a hyperendemic pattern of transmission. During the 2000s the highest incidence was reported in the group of age between 5 – 14 years.. Since 2006, the four serotypes of the virus have been in many municipalities of the country.
Ocazionez et al., 2006 [17]	North-eastern region of Colombia: Bucaramanga city, Santander department	Longitudinal serotype-specific surveillance and cross-sectional study	Surveillance study: 1998–2004 Cross-sectional study: May 2003-December 2004	Surveillance study: 1,452 acute serum samples from febrile patients	Mean age reported by year	Study of temporal distribution of dengue (DEN) virus serotypes in relation to dengue incidence, infection pattern, and severity of disease. Predominance of DENV-1 in 1998 and re-introduction and predominance of DENV-3 in 2001-2003 coincided with dengue outbreaks. DHF was more frequent in DENV-2 infected patients than in DENV-3 infected patients. DENV-4 was isolated in 2000-2001 and 2004.There was an annual increase of primary dengue infections (from 13.7 to 81.4%) that correlated with frequency of DEN-3 ($r = 0.83$; $P = 0.038$).
Mendez et al., 2010 [18]	n/a	Phylogenetic study	1978–2005	n/a	n/a	Clinical samples were collected between 1978 and 2007 from different localities; representing most viruses circulating in Colombia during the last 30 years. Only one genotype of DENV 1 has been circulating since the first epidemic reports on the continent, although two different lineages have been evolving since the early 1990′s.
Instituto Nacional De Salud [23]	Orinoquia region, Colombia Guainía region (capital Puerto Inírida)	Surveillance data	2010 (to week 52)	157,152 cases	Not specified	Notification of dengue cases to the Instituto Nacional de Salud (SIVIGILA)

Citation	Region/geographical area	Study design/data gathering method	Data period: Date range/year	If study: No. patients/ population studied (M:F)	Age range	Summary of data presentation or results/conclusion
Mera et al., 2003 [24]	Departments of Guaviare, Putumayo, Caquetá and North Santander	Surveillance data analysis	1998–2002	81,831 cases of classic dengue were reported in 2002	Not specified	Regional surveillance system data on dengue diseases to inform the dengue situation in Colombia
Rojas-Álvarez, 2008 [25]	Nationwide data	Surveillance data	2007 (through to week 52)	43,541 patients (52%:48%)	Classic dengue: 1–98y, average age 22.5y, median age 17y, modal age 10y. DHF: 1–98 years, average age 21y, median age 15y, modal age 7y.	This report is based on data recorded at the SIVIGILA through 2007.The cumulative total of reported cases to SIVIGILA during 2007 was 43,541 cases of dengue, 89% of which were classic dengue (38,895) and 11% hemorrhagic dengue (4.646). 45% of cases of dengue fever occurred in the 15-45y age group (5-14y: 29%); 51% of DHF cases were aged <15y. Compared with 2006 an increase of 12% of dengue cases was observed.
Rojas-Álvarez, 2010 [26]	Nationwide data	Surveillance data	2009 (through to week 52)	55,592 cases of classic dengue and 9,137 cases of DHF were reported.(classic dengue = 54%:46%; DHF = 51%:49%)	Classic dengue: 1mo–98y (average 21.3y, median 16y, mode 9) DHF: 1mo–97y (average 17.6y, median 11y, mode 8y)	This descriptive report presents data gathered by the national surveillance system (SIVIGILA) for 2009. A significant increase in the cases of documented dengue patients during the 2009 year (n=51,543), was observed compared with 2008 (n=39,814) and an increase (1.9 fold) in the number of deaths compared to the same period in 2008 (n=44 vs. n=23).
Instituto Nacional De Salud [27]	Whole country	Surveillance data	2009 (to week 52)	41,819 cases DF; 6,554 cases DHF	Not specified	Notification of dengue cases to the Instituto Nacional de Salud (SIVIGILA)
Rojas-Álvarez and Padilla 2010 [28]	Nationwide data	Surveillance data analysis<br><br>[Conference presentation abstract]	November 2009 to August 2010	134,231 cases of dengue ((93% without warning signs)	13.5y (mean age for the cases of dengue without warning signs)	Of the 134,231 cases of dengue reported to SIVIGILA, 124,998 (93%) were cases of dengue without warning signs; 9,233 (7%) were cases of severe forms of dengue. Laboratory confirmation was obtained in 43% of the cases of dengue without warning signs and 48% of the cases of severe dengue. The incidence of the disease to August 2010 was 537/100.000. 60% of the cases were reported in 13 of 32 departments of the country. The isolated serotypes where: DENV-1 (41%), DENV-2 (44%), DENV-3 (12%) and DENV-4 (3%).

Citation	Region/geographical area	Study design/data gathering method	Data period: Date range/year	If study: No. patients/ population studied (M:F)	Age range	Summary of data presentation or results/conclusion
Instituto Nacional De Salud [29]	Nationwide data	Surveillance data	2011 (to week 52)	32,755 cases	Not specified	Notification of dengue cases to the Instituto Nacional de Salud (SIVIGILA)
Rojas-Álvarez, 2011 [30]	Nationwide data	Surveillance data	2010 (through to week 52)	150,941 cases reported (classic dengue: 53%:47%; serious dengue: 51%:49%	Classic dengue: average age 13y, median age 14–15y<br>Serious dengue: average age 21.6y, median age 15y, mode 8y	This report is based on data recorded at the SIVIGILA through 2010.As of epidemiological week 48 of the 150,941 cases reported 9,368 (6%) were severe dengue. There were 67,825 (48%) cases of dengue and 5,031 (54%) of severe dengue confirmed. Median of 3,144 cases a week notified. The age group most affected were those <15y (44%); 65% of cases of severe dengue occurred in the <15y group. The need for hospitalization was 32% dengue and severe dengue than 79%. A significant increase in cases was observed during 2010 compared with 2009 and there was a significant increase (4.1 times) in the number of dengue deaths compared to the same period in 2009.
Pérez, 2010 [31]	Nationwide data	Surveillance data	2011 (up to week 52)	30,694 reported cases (Dengue: 42%:58%; Severe dengue: 55%: 45%)	1mo–98y (Mean: 26y; Median age: 21y)	Regional sentinel surveillance data on dengue diseases to inform the dengue situation in Colombia
Instituto Nacional De Salud, 2012 [32]	Nationwide data	Surveillance data	2012 (up to week 52)	53,879 reported cases (Dengue: 54.7%:45.3%; Severe dengue: 52.8%:47.2%)	Mean age: 23y<br>Median age: 16y<br>Severe dengue: mean: 17y<br>Median: 13y	Data reported to SIVIGILA through epidemiological week 51, 2012
Rodríguez et al., 2006 [33]	Palmira, Valle del Cauca	Descriptive study	2001–2004	Regional population	<1y to >60y	This study used data from the Public Health monitoring system in Palmira. A reduction of 88.2% in reports of classic dengue was observed from 2001 to 2004; this reduction was greater in the 15-44y age group (92.2%); in 5-14y and 45-59y groups the reductions were 89.8% and 88.2%, respectively. The authors attributed this decrease in reporting to a corresponding Municipal Health training programme for the control and prevention of dengue in the first half of 2003.

Citation	Region/geographical area	Study design/data gathering method	Data period: Date range/year	If study: No. patients/ population studied (M:F)	Age range	Summary of data presentation or results/conclusion
Pacheco, 2004 [34]	Guaviare department	Sentinel surveillance	Jan– Sep 2004 (37 weeks)	1,220 febrile patients(1,049 sera processed for dengue IgM testing) (62.85%:37.15%)	2–90 years (median 27y)	1,049 sera were processed for dengue IgM tests in the Public Health Laboratory; of these, 38.9% (n = 409) were positive; in these cases were 38.4% women and 61.6% were men (p <0.01).
Rojas-Álvarez, 2008 [35]	Puerto Inírida, Guainía	Observational, descriptive cross-sectional study	2007–2008	1,241 probable dengue cases (39.2%: 60.8%):	2–76y (SD 17.3, median 31.7)	The aim of this study was to establish the aetiology of an outbreak of fever, and develop guidelines for its adequate prevention and control. This outbreak of dengue fever started in week 49 of 2007 and lasted until the 12th week of 2008; however, dengue was not suspected and notification to the Department of Health was not made until week 7, 2008. Consequently, therefore individual control measures to reduce the outbreak were not instigated early.
Salgado et al., 2007 [36]	Neiva, southern Colombia	Descriptive, retrospective study	Jan–June 2004	105 (46:59)	0–<13y	Clinical and para-clinical data were recorded and analysed: 105 children were diagnosed with either DHF (87.6%) or DF (12.4%); 67% were aged <5y. 83% of the children were admitted to hospital during the first 6 days of the disease; dengue shock syndrome was diagnosed in 20%..
Mendez et al., 2006 [37]	Focus was on four cities in Valle del Cauca: Cali, Palmira, Tuluá, and Buenaventura (south-western Colombia)	Prospective epidemiology study	November 2002 (during dengue epidemic) to March 2004 (after epidemic)	3189 (1080:2109)	7–20y	Infection in humans was diagnosed by virus isolation using the C636 cell line of Ae. albopictus and by immunofluorescent analysis using serotype-specific monoclonal antibodies. Adult mosquito and larvae house indexes were not associated with increased burden of disease. The only entomologic indicator related to dengue infection in humans was the pooled infection rate of mosquitoes. Infection rates showed significant differences between the two periods of the study (epidemic 10.68, 95% CI 7.04–15.62 and non-epidemic 6.15, 95% CI 3.46–10.19).

Citation	Region/geographical area	Study design/data gathering method	Data period: Date range/year	If study: No. patients/ population studied (M:F)	Age range	Summary of data presentation or results/conclusion
Ocazionez et al., 2007 [38]	Santander department	Descriptive retrospective study	1998–2004	1,545 febrile patients	4–73y	Dengue infection was confirmed by IgM ELISA and the virus was isolated in C6/36 cells. The re-introduction of DENV-3 in 2001 coincided with an epidemic. The was a greater association of secondary infection with DENV-2 than with DEN-3 (56,8 % cf 15,7 %; p<0.001). Change in relative dengue virus serotype abundance was associated with changed infection pattern and DHF frequency.
Rangel et al., 2008 [39]	North-central Santander department	Phylogenetic study	18 Jan 2007 to 11 Oct 2008	680 serum samples from suspected dengue patients	Not specified	Study of the relative abundance of DENV-3 in relation to the incidence of DHF. In 2007–2008 the proportion of DENV-3 declined compared with 2002–2004 (28.3% vs.87.8%), whereas DENV-1 and DENV-2 proportions increased (54.7% vs. 2.7% and 16.9% vs. 5.4%, respectively). This change coincided with an increase of DHF compared with the period of the highest DENV-3 incidence (25.9% vs. 4.6%).
Ospina et al., 2010 [40]	Medellín	Phylogenetic study	2002–2007	18 viruses obtained for study (from 18 patients)	9–69y	An unexpected co-circulation of twoDENV-3 subtype III variants was found during the study period. An analysis of DENV-3 viruses isolates from other South American regions revealed three different subtype III lineages, derived from independent sources.
Ospina, 2004 [41]	Antioquia	Epidemiological surveillance study	1980–2003	Regional population	Not specified	Data from Antioquia Region Epidemiological Surveillance Program: Notified cases between 1980 and 2003; incidence of dengue in Antioquia ranged between 18.5 and 183 cases per 100,000 population.
Ocazionez et al., 2006 [48]	North-eastern region of Colombia: Bucaramanga city, Santander department	Longitudinal serotype-specific surveillance and cross-sectional study	S'v study: 1998–2004<br>Cross-sectional study: May 2003-December 2004	Surveillance study: 1,452 acute serum samples from febrile patients	Mean age reported by year	In 146 cases with complete and reliable clincial and laboratory data, 53 (24.5%) with DEN-2 developed DHF, 3 of whom died; 71 (11.2%) with DEN-3 developed DHF. The infection pattern was evaluated in 596 laboratory-positive dengue cases using an IgG ELISA, and PRNT test. The dengue incidence was documented by the local health authority. Study of the temporal distribution of DENV serotypes. DENV-3 re-introduction and predominance in 2001-2003 coincided with outbreaks. Predominance of DEN-2 in 2000-2001

Citation	Region/geographical area	Study design/data gathering method	Data period: Date range/year	If study: No. patients/ population studied (M:F)	Age range	Summary of data presentation or results/conclusion
Rangel et al., 2008 [49]	North-central Santander department	Phylogenetic study	18 Jan 2007 to 11 Oct 2008	680 serum samples from suspected dengue patients	Not specified	Study of the relative abundance of DENV-3 in relation to the incidence of DHF in a Colombian endemic area. In 2007–2008 the proportion of DENV-3 declined compared with 2002–2004 (28.3% vs.87.8%), whereas DENV-1 and DENV-2 proportions increased (54.7% vs. 2.7% and 16.9% vs. 5.4%, respectively). This change coincided with an increase of DHF compared

Sources included during the data extraction process but not providing information cited in the text						
Osorio et al., 2009 [A]	Medellín	Case study<br><br>[Conference presentation abstract]	20 March 2008 to 20 March 2009	781 febrile patients	1mo–89y (median: 3y)	The study aimed to determine the proportion of febrile illness due to dengue among patients presenting to ambulatory clinics. Laboratory confirmation of dengue infection was obtained in 66 patients (26 by RT-PCR; 40 by MAC ELISA. 8 patients required hospitalization. Only 2 patients presenting with fever were diagnosed with dengue by the treating physician. The results suggest that the burden of dengue in Medellin may be greater than that indicated by surveillance data.
González et al., 2008 [B]	Bucaramanga	Retrospective observational study	2006–2007	Cohort = 328 dengue inpatients (163 [49.7%]:165 [50.3%])	3 months to 80y (median 25y)	WHO criteria for dengue fever met in 299 patients (91.2%) Age was the most determinant factor of severity in dengue inpatients. Children presented the typical symptoms of dengue less frequently, but demonstrated a greater proportion of ascites, pleural effusion and bleeding, and a higher risk of developing respiratory distress (RR=3.59, 95%CI 1.3-9.9, p<0.014) and hypotension (RR=10.77, 95%CI 5.56-20.86, p<0.001).

A. Osorio J, Beatty M, Goez Y, Restrepo L (2009) Frequency of dengue fever among febrile patients presenting to an urban hospital in Medellin, Colombia: study results.Poster presented at: American Society of Tropical Medicine and Hygiene 58th Annual Meeting, 18-22 November 2009 Washington, D.C., USA. Abstract no. 794

B. González A, Martínez R, Villar L (2008) [Clinical evolution of dengue in hospitalized patients]. Biomédica 28: 531-543
