# Supplementary figures and images for: Epidemiological Trends of Dengue Disease in Colombia (2000-2011): A Systematic Review
Source: PLoS Negl Trop Dis. 2015 Mar 19;9(3):e0003499. doi: 10.1371/journal.pntd.0003499 (PMC4366106; doi:10.1371/journal.pntd.0003499)

**Figure S1. Departments of Colombia.**

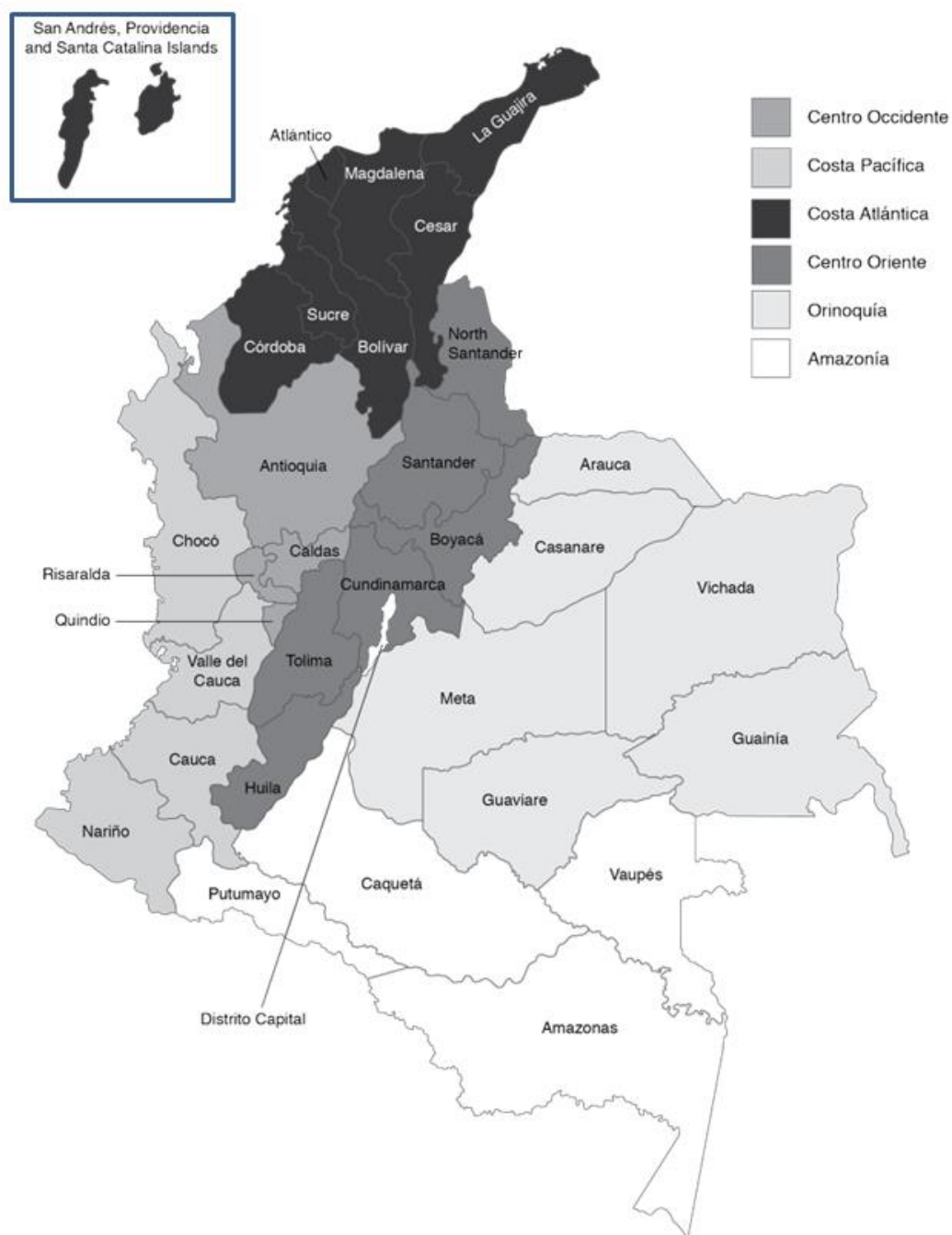

Supplement: S1 Fig — (PDF) [file pntd.0003499.s004.pdf]
